# Pre-exposure prophylaxis with hydroxychloroquine for high-risk healthcare workers during the COVID-19 pandemic: A structured summary of a study protocol for a multicentre, double-blind randomized controlled trial

**DOI:** 10.1186/s13063-020-04621-7

**Published:** 2020-07-29

**Authors:** Berta Grau-Pujol, Daniel Camprubí, Helena Marti-Soler, Marc Fernández-Pardos, Caterina Guinovart, Jose Muñoz

**Affiliations:** 1 Barcelona Institute for Global Health (ISGlobal), Hospital Clínic - Universitat de Barcelona, Barcelona, Spain; 2 Fundación Mundo Sano, Argentina, Spain; 3 Centro de Investigação em Saúde da Manhiça, Maputo, Mozambique

**Keywords:** COVID-19, Randomised controlled trial, protocol, pre-exposure, prophylaxis, hydroxychloroquine, healthcare workers, Spain, double-blinded

## Abstract

**Objectives:**

The aim of this study is to assess the efficacy of the use of pre-exposure prophylaxis (PrEP) with hydroxychloroquine against placebo in healthcare workers with high risk of severe acute respiratory syndrome coronavirus 2 (SARS-CoV-2) infection in reducing their risk of coronavirus disease 2019 (COVID-19) disease during an epidemic period.

As secondary objectives, we would like to: i) assess the efficacy of the use of PrEP with hydroxychloroquine against placebo in healthcare workers with high risk of SARS-CoV-2 infection in reducing their risk of exposure to SARS-CoV-2 (defined by seroconversion) during an epidemic period, ii) evaluate the safety of PrEP with hydroxychloroquine in adults, iii) describe the incidence of SARS-CoV-2 infection among healthcare workers at high risk of SARS-CoV-2 infection, iv) identify clinical, analytical and microbiological predictors of COVID-19 among healthcare workers at high risk of SARS-CoV-2 infection, v) set up a repository of serum samples obtained from healthcare workers at high risk of SARS-CoV-2 infection for future research on blood markers to predict SARS-CoV-2 infection.

**Trial design:**

Multicentre double-blind parallel design (ratio 1:1) randomized controlled clinical trial.

**Participants:**

Approximately 440 healthcare workers of four Spanish hospitals (Hospital Clínic of Barcelona, Hospital de la Santa Creu i Sant Pau of Barcelona, Hospital Plató of Barcelona, Hospital General de Granollers, Barcelona) will be recruited. Participants are considered to be at high-risk of SARS-CoV-2 infection due to their frequent contact with suspected and confirmed cases of COVID-19.

For eligibility, healthcare workers with 18 years old or older working at least 3 days a week in a hospital with both negative SARS-CoV-2 polymerase chain reaction (PCR) assays and serological COVID-19 rapid diagnostic tests (RDT) are invited to participate. Participants with any of the following conditions are excluded: pregnancy, breastfeeding, ongoing antiviral, antiretroviral or corticosteroids treatment, chloroquine or hydroxychloroquine uptake the last month or any contraindication to hydroxychloroquine treatment.

**Intervention and comparator:**

Eligible participants will be allocated to one of the two study groups:

- Intervention group (PrEP): participants will receive the standard of care and will take 400mg of hydroxychloroquine (2 tablets of 200 mg per Dolquine® tablet) daily the first four consecutive days, followed by 400 mg weekly for a period of 6 months.
- Control group: participants will receive placebo tablets with identical physical appearance to hydroxychloroquine 200 mg (Dolquine®) tablets following the same treatment schedule of the intervention gr*ou*p.

Both groups will be encouraged to use the personal protection equipment (PPE) for COVID-19 prevention according to current hospital guidelines.

**Main outcomes:**

The primary endpoint will be the number of confirmed cases of a COVID-19 (defined by a positive PCR for SARS-CoV-2 or symptoms compatible with COVID-19 with seroconversion) in the PrEP group compared to the placebo group at any time during the 6 months of the follow-up in healthcare workers with negative SARS-CoV-2 PCR and serology at day 0.

As secondary endpoints, we will obtain: i) the SARS-CoV-2 seroconversion in the PrEP group compared to placebo during the 6 months of follow-up in healthcare workers with negative serology at day 0; ii) the occurrence of any adverse event related with hydroxychloroquine treatment; iii) the incidence of SARS-CoV-2 infection and COVID-19 among healthcare workers in the non-PrEP group, among the total of healthcare workers included in the non-PrEP group during the study period; iv) the risk ratio for the different clinical, analytical and microbiological conditions to develop COVID-19; v) a repository of serum samples obtained from healthcare workers confirmed COVID-19 cases for future research on blood markers to predict SARS-CoV-2 infection.

**Randomisation:**

Participants meeting all eligibility requirements will be allocated to one of the two study arms (PrEP with hydroxychloroquine or non-PrEP control group) in a 1:1 ratio using simple randomisation with computer generated random numbers.

**Blinding (masking):**

Participants, doctors and nurses caring for participants, and investigators assessing the outcomes will be blinded to group assignment.

**Numbers to be randomised (sample size):**

Each intervention group will have 220 participants, giving a total of 440 participants.

**Trial Status:**

The current protocol version is 1.5, 2^nd^ of June 2020. Two hundred and seventy-fiveparticipants were recruited and completed first month follow-up until date. The estimated sample size could not be reached yet due to the declining national epidemic curve. Thus, 275 is the total number of participants included until date. The study has been suspended (26^th^ of June) until new epidemic curve occurs.

**Trial registration:**

This trial was registered on April 2^nd^ 2020 at clinicaltrials.gov with the number NCT04331834.

**Full protocol:**

The full protocol is attached as an additional file, accessible from the Trials website (Additional file 1). In the interest in expediting dissemination of this material, the familiar formatting has been eliminated; this Letter serves as a summary of the key elements of the full protocol.

## Supplementary information

**Additional file 1.**

## Data Availability

Data will be available from the author on reasonable request (jose.munoz@isglobal.org).

