# Supplementary material for: Pre-exposure prophylaxis with hydroxychloroquine for high-risk healthcare workers during the COVID-19 pandemic: A structured summary of a study protocol for a multicentre, double-blind randomized controlled trial
Source: Trials. 2020 Jul 29;21:688. doi: 10.1186/s13063-020-04621-7 (PMC7388426; doi:10.1186/s13063-020-04621-7)
Supplement: Supplementary file 1 — Additional file 1. [file 13063_2020_4621_MOESM1_ESM.pdf]

# PRE-EXPOSURE PROPHYLAXIS WITH HYDROXYCHLOROQUINE FOR HIGH-RISK HEALTHCARE WORKERS DURING THE COVID-19 PANDEMIC (PrEP_COVID): A MULTICENTRIC, DOUBLE-BLINDED RANDOMIZED CONTROLLED TRIAL.

## 1- GENERAL INFORMATION

1.1. **Protocol title:** Pre-exposure prophylaxis with Hydroxychloroquine for high-risk healthcare workers during the COVID-19 pandemic (PrEP_COVID)

1.2. **Protocol ID number/date:** PrEP_COVID_V1.5_02nd June 2020

1.3. **Sponsor**

ISGlobal - Barcelona Institute for Global Health
c/ Rosselló 132, 4 – 1. 08036. Barcelona. Spain.

1.4. **Researchers**


Principal investigator (PI)

Jose Muñoz, MD MSc PhD

Consultant. International Health Department, Hospital Clínic. Associate Research Professor-ISGlobal

Co-investigators
Daniel Camprubí. International Health Department. Hospital Clínic Barcelona.
Alejandro Almuedo. International Health Department. Hospital Clínic Barcelona.
Sara Ajanovic. ISGlobal - Barcelona Institute for Global Health.
Sophie Jullien. ISGlobal - Barcelona Institute for Global Health.
Felipe García. Infectious Diseases Department. Hospital Clínic Barcelona.
Giuseppe Barilaro. ISGlobal - Barcelona Institute for Global Health.
Pere Domingo. Hospital de Sant Pau, Barcelona
Jordi Altes. Hospital Plató, Barcelona
Noemí Busquets. Hospital General de Granollers.

Key Personnel
Caterina Guinovart. ISGlobal - Barcelona Institute for Global Health.
Berta Grau. ISGlobal - Barcelona Institute for Global Health.
Helena Martí. ISGlobal - Barcelona Institute for Global Health.
Mª Angeles Marcos. Microbiology Department. Hospital Clínic Barcelona.3
Dolors Soy. Pharmacology Department. Hospital Clínic Barcelona.
Elisabet Ferrer. International Health Department. Hospital Clínic Barcelona.

Isabel Vera. International Health Department. Hospital Clínic Barcelona.
Montse Roldan. International Health Department. Hospital Clínic Barcelona.
Marc Fernández Pardos. ISGlobal - Barcelona Institute for Global Health.

## 2. BACKGROUND:

### 2.1. State of the art and preliminary data:

In December 2019, the Wuhan Municipal Health Committee (Hubei province, China) informed about an outbreak of viral pneumonia of unknown aetiology (1). Only a few weeks later, a new RNA virus from the family *Coronaviridae*, further called "severe acute respiratory syndrome coronavirus 2 (SARS-CoV-2)", was identified as the causative agent of the outbreak (2). The human disease caused by SARS-CoV-2 has been designated COVID-19. From then onwards, more than 130,000 confirmed cases and almost 5,000 deaths have been reported (3). Since the onset of the outbreak, SARS-CoV-2 has spread to 122 countries, affecting Asia, Africa, Europe and America, and has been declared a pandemic by the World Health Organization (3).

Besides efforts to mitigate COVID-19 impact in terms of morbidity and mortality, prevention of secondary cases is a key point to prevent the spread of the disease. Recent estimates suggest that secondary attack rate in household contacts of COVID-19 cases ranges from 3-10% (4). Prevention of healthcare professionals' infection is crucial for the management of a pandemic such as COVID-19. Pre-exposure prophylaxis (PrEP) has already been demonstrated to be useful in preventing other diseases (5) infections in the context of high risk exposure. On the other hand, recent studies showed that chloroquine can effectively inhibit SARS-CoV-2 (6) and that hydroxychloroquine has a better *in vitro* antiviral activity than chloroquine (7). In addition, it is safer than chloroquine and is suitable for pregnant women. Hydroxychloroquine has been suggested to be used as prophylaxis since inhibits the cytokine storm by suppressing T cell activation and this could prevent SARS-CoV-2 infection and COVID-19 progression. However, usefulness of PrEP with hydroxychloroquine in preventing COVID-19 among healthcare professionals has not been evaluated yet.

### 2.2. Rationale:

## 3. HYPOTHESIS AND OBJECTIVES:

### 3.1. Hypothesis

Pre-exposure prophylaxis (PrEP) with hydroxychloroquine would be an effective measure for preventing COVID-19 among healthcare workers at high risk of SARS-CoV-2 infection due to their frequent contact with suspected and confirmed cases of COVID-19.

## 3.2. **Objectives:**

### 3.2.1. Primary objective:

1) To compare the efficacy of the use of PrEP with hydroxychloroquine against placebo in healthcare workers with high risk of SARS-CoV-2 infection in reducing their risk of COVID-19 disease during an epidemic period.

### 3.2.2. Secondary objectives:

1) To assess the efficacy of the use of PrEP with hydroxychloroquine against placebo in healthcare workers with high risk of SARS-CoV-2 infection in reducing their risk of exposure to SARS-CoV-2 (defined by seroconversion) during an epidemic period.
2) To evaluate the safety of PrEP with hydroxychloroquine in adults.
3) To describe the incidence of SARS-CoV-2 infection among healthcare workers at high risk of SARS-CoV-2 infection.
4) To identify clinical, analytical and microbiological predictors of COVID-19 among healthcare workers at high risk of SARS-CoV-2 infection.
5) To set up a repository (biobank) of serum samples obtained from healthcare workers at high risk of SARS-CoV-2 infection for future research on blood markers to predict SARS-CoV-2 infection.

## 3.3. **Endpoints:**

### 3.3.1. Primary endpoint:

1) Confirmed cases of a COVID-19 (defined by a positive PCR for SARS-CoV-2 or symptoms compatible with COVID-19 with seroconversion) in the PrEP group compared to the placebo group at any time during the 6 months of the follow-up in healthcare workers with negative PCR for SARS-CoV-2 at day 0.

### 3.3.2. Secondary endpoints:

1) SARS-CoV-2 seroconversion in the PrEP group compared to placebo in during 6 months of follow-up in healthcare workers with negative serology at day 0.
2) The occurrence of any adverse event related with hydroxychloroquine treatment. We will compare the incidence of clinical and/or laboratory adverse events in the hydroxychloroquine PrEP group and the non-PrEP group.
3) Incidence of SARS-CoV-2 infection and COVID-19 among healthcare workers will be estimated by the number of healthcare workers diagnosed with COVID-19 in the

non-PrEP group, among the total of healthcare workers included in the non-PrEP group during the study period.

4) Risk ratio for the different clinical, analytical and microbiological conditions to develop COVID-19.
5) A repository (biobank) of serum samples obtained from healthcare workers confirmed COVID-19 cases for future research on blood markers to predict SARS-CoV-2 infection.

# 4. METHODS:

## 4.1. Study design:

Multicentric double-blinded randomized controlled clinical trial

## 4.2. Study groups:

Eligible participants will be allocated to one of the 2 study groups:

- Intervention group (PrEP): participants will receive the standard of care and will take hydroxychloroquine (200 mg per tablet: Dolquine®) with the following dosage:
  - day 0: 400 mg (2 tablets)
  - day 1: 400 mg (2 tablets)
  - day 2: 400 mg (2 tablets)
  - day 3: 400 mg (2 tablets)
  - weekly: 400 mg (2 tablets) for a period of 6 months
- Control group: participants will receive placebo tablets with identical physical appearance to hydroxychloroquine 200 mg (Dolquine®)

Standard of care for COVID-19 is currently considered EPI B, following currently active hospital guidelines.

## 4.3. Study population

Approximately 440 healthcare workers of the Hospital Clinic Barcelona, Hospital de Granollers, Hospital de Sant Pau and Hospital Plató will be recruited. Participants will be considered as high-risk of SARS-CoV-2 infection due to their frequent contact with suspected and confirmed cases of COVID-19.

### 4.3.1. Inclusion criteria

- Age ≥ 18 years
- Negative PCR and negative serology at day 0
- Healthcare worker at any of the trial sites

- Female participants of reproductive age: negative for pregnancy test
- Willing to participate in the study
- Able to sign the informed consent form

#### 4.3.2. Exclusion criteria

- Age <18 years
- Pregnancy or breastfeeding
- Ongoing antiviral or antiretroviral treatment or HIV positive
- Ongoing anti-inflammatory treatment (corticosteroids)
- Ongoing or previous treatment (1 month) with chloroquine or hydroxychloroquine
- Confirmed case of SARS-CoV-2 infection (positive PCR) at day 0
- Positive serology for SARS-CoV-2 infection at day 0
- Impossibility of signing the informed consent form
- Rejection of participation
- Working less than 3 days a week in the Hospital.
- Any contraindication for hydroxychloroquine treatment (9):
  - Hydroxychloroquine hypersensitivity or 4-aminoquinoline hypersensitivity
  - Retinopathy, visual field or visual acuity disturbances
  - QT prolongation, bradycardia (<50 bpm), ventricular tachycardia, other arrhythmia, as determined on day 0 ECG or medical history
  - Previous myocardial infarction
  - Potassium < 3 mEq/L or AST or ALT > 5 upper normal limit, as determined on day 0 blood test
  - Glomerular clearance < 10ml/min
  - Myasthenia gravis
  - Porphyria
  - Previous history of severe hypoglycaemia
  - Ongoing treatment with: antimalarials, antiarrhythmic, tricyclic antidepressants, selective serotonin reuptake inhibitors, natalizumab, quinolones, macrolides, agalsidase alfa and beta.

### 4.4. Study location

This study will be hospital-based and will be conducted at Hospital Clinic of Barcelona, Hospital de Sant Pau, Hospital Plató and Hospital de Granollers Spain.

## 5. STUDY PROCEDURES

### 5.1. Screening visit

All healthcare workers from Hospital Clinic, Hospital de Sant Pau, Hospital Plató and Hospital de Granollers will be invited to participate. Specifically, all healthcare workers with a negative PCR at the last Hospital Clinic routine SARS-CoV-2 screening will be also invited to participate. Study personnel will inform them about the option to enrol in this study.
On the day of the screening visit, the following procedures will be performed:

- Nasopharyngeal swab to conduct PCR to detect SARS-CoV-2.
- Detailed evaluation of previous medical history, allergies, current treatment, symptoms and physical examination (Appendix 2).
- 12 mL of venous blood will be taken for the following determinations:
  - Blood test (WBC, lymphocyte populations, haemoglobin, platelets, liver and kidney function, bilirubin, LDH, PCR)
  - Rapid diagnostic serology test (RDT) for SARS-CoV-2 (IgM and IgG)
  - HIV serology
  - Pregnancy test (women of reproductive age 18 - 55 years old)
- 10 mL of venous blood to store two serum samples:
  - 2mL to perform a serology test to detect SARS-CoV-2 Immunoglobulins M and G (IgM and IgG)
  - 2mL for biobank storage (for further research on diagnostics/biomarkers). 0
- 1 mL of whole blood will be collected for hydroxychloroquine PK analysis.
- One strip ECG (V2) to evaluate cardiac rhythm
- Informed consent (Appendix 1) will be obtained from all healthcare workers willing to participate who fulfil the inclusion and exclusion criteria

In case RDT serological test is negative at screening visit but the serum sampled stored to detect SARS-CoV-2 Immunoglobulins M and G (IgM and IgG) is positive, the participant will be excluded from the analysis.

Participants fulfilling inclusion criteria and none of the exclusion criteria will be scheduled for recruitment visit.

Participants who fail to meet the inclusion and exclusion criteria will not, under any circumstances, be enrolled or receive study treatment. If any participant is enrolled by error, they will be withdrawn from the study, the PI notified and records kept. Any participant incorrectly enrolled who started the intervention, will be followed up for AE.

### 5.2. Recruitment and randomization visit (day 0)

Participants meeting all eligibility requirements will be allocated to one of the two study arms (PrEP with hydroxychloroquine or non-PrEP control group) in a 1:1 ratio. A real-time computer-generated randomization procedure will be used.

At recruitment visit, a numbered case record form (CRF, Appendix 2) will be filled out for all potential participants.
The first 2 tablets will be taken directly observed at the baseline visit. The rest of the tablets of the first month will be provided to the participant during that visit.

### 5.3. Blinding

The study will be double blinded.

### 5.4. Study participant flowchart

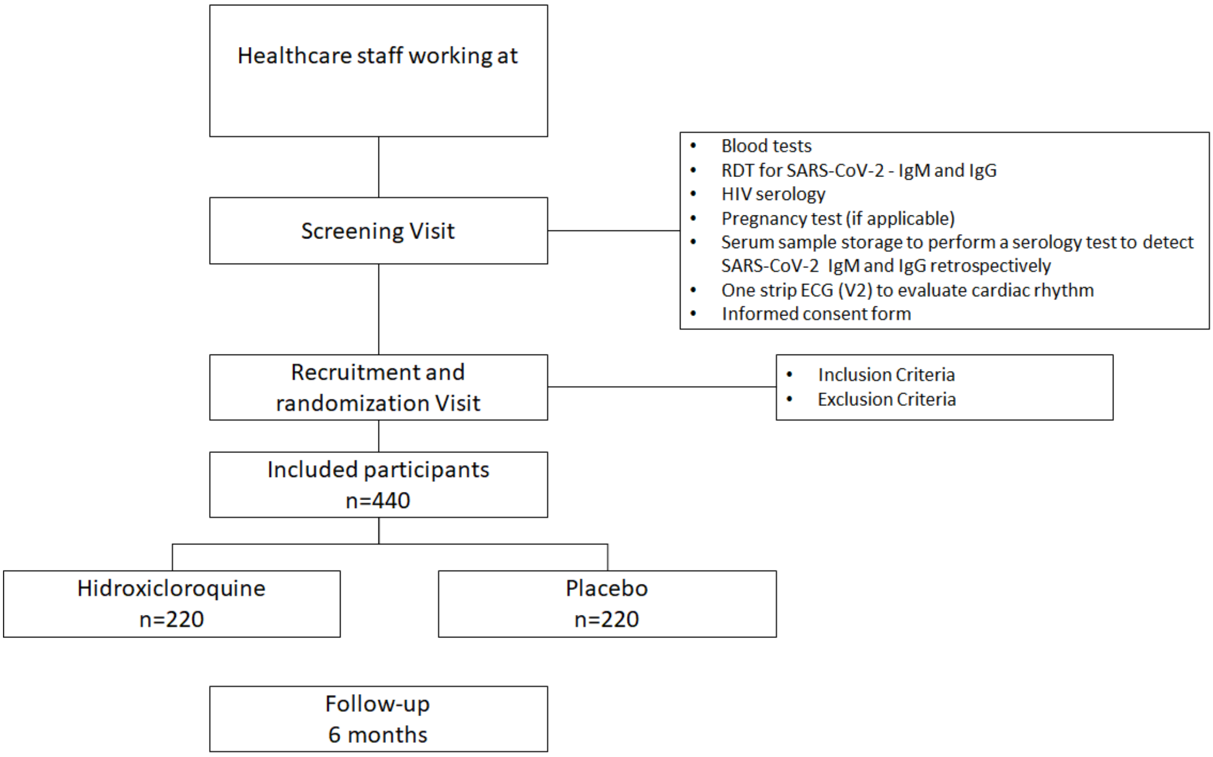


*Figure 1 Participant flowchart*

### 5.5. Follow-up

Regardless of their group of allocation, all participants will undergo the same procedures and will be followed-up monthly during 6 months. We will conduct active and passive surveillance to all of them.

Active surveillance to each participant will be conducted monthly, when a standardized CRF will be filled out by the study team medical doctor conducting the visit. Follow-up visits will include:

1. Detailed evaluation of symptoms and physical examination (to either detect past and current symptoms and signs related with COVID-19 or past and current adverse events of hydroxychloroquine treatment since the previous visit)
2. Known close contacts with suspected and/or confirmed COVID-19 cases
3. Assessment of compliance with PrEP and other preventive measures, including the number of weeks during which they have been managing COVID-19 patients.
4. 12 mL of venous blood will be taken at month 1, 3 and 6 visits for the following determinations: WBC, lymphocyte populations, haemoglobin, platelets, liver and kidney function, bilirubin, LDH, PCR
5. 10 mL of venous blood to store two serum samples:
    - 2mL to perform a serology test to detect SARS-CoV-2 Immunoglobulins M and G (IgM and IgG)
    - 2mL for biobank storage (for further research on diagnostics/biomarkers).
6. One strip ECG (V2) to evaluate cardiac rhythm

In addition, on day 0, 3, 10, 31 (scheduled visit (M1)) and 61 (scheduled visit (M2)), 1 mL of whole blood will be collected to evaluate hydroxychloroquine concentrations. At every visit, the study medical doctor will also check for symptoms or possible adverse events.

Passive surveillance for each participant will be also conducted. A medical doctor will be available by phone 24 hours a day during the study period. All participants will be allowed to call this number in case of presenting any adverse event or symptom. In that case, a standardized CRF will be filled out to collect the information. A chest X-ray and a nasopharyngeal swab will be performed to all those participants presenting with fever or respiratory symptoms.

### 5.6. Visit schedule

| | Screening visit | Recruitment visit (Day 0) | Day 3 | D10 | M1 | M2 | M3 | M4 | M5 | M6 |
|---|---|---|---|---|---|---|---|---|---|---|
| Informed consent | X | | | | | | | | | |
| CRF | X | | X | X | X | X | X | X | X | X |
| Treatment group allocation | | X | | | | | | | | |
| Adverse events evaluation | | X | X | X | X | X | X | X | X | X |
| General blood tests | X | | | | X | X | X | X | X | X |
| HIV serology | X | | | | | | | | | |
| Pregnancy test | X | | | | | | | | | |

| | | | | | | | | | | |
|---|---|---|---|---|---|---|---|---|---|---|
| ECG | X | | | | X | X | X | X | X | X |
| Serology SARS-CoV-2 | X | | | | X | X | X | X | X | X |
| Nasopharyngeal swab | X | | | | | | | | | |
| COVID-19 serology RDT | X | | | | X | | | | | |
| Serum (biomarkers) | X | | | | X | X | X | X | X | X |
| PK analysis | X | | X | X | X | X | | | | |

*Tabla 1 Visit schedule*

## 5.7. **Timelines for the completion of the clinical trial**

| | Mar 20 | Apr 20 | May 20 | Jun 20 | Jul 20 | Aug 20 | Sep 20 | Oct 20 |
|---|---|---|---|---|---|---|---|---|
| Site selection | | | | | | | | |
| Protocol submission and authorisation | | | | | | | | |
| Screening | | | | | | | | |
| Recruitment | | | | | | | | |
| Drug administration | | | | | | | | |
| Follow-up | | | | | | | | |
| Data analysis | | | | | | | | |
| Publication and dissemination | | | | | | | | |

## 5.8. **Biobank**

De-identified samples will be indefinitely stored at the Hospital Clinic de Barcelona biobank for future use in other scientific projects related to SARS-CoV-2 infection. Samples will be stored at the “Colección de Patología Importada”, registered at the Hospital Clínic-IDIBAPS Biobank and in the National Biobank Registry with code number C.0002610. Those coded data and samples could be sent abroad to other research institutions for usage in research projects if necessary. In this case it will be assured that identical standards to the ones applicable for this research project are fulfilled. Patients will sign a separated informed consent if they agree to the storage of their samples in the biobank (Appendix 1) and the shipment of the samples to other institutions.

# 6. STATISTICAL ANALYSIS

## 6.1. <u>Sample size calculation</u>:

With an expected incidence of 10% of COVID-19 in healthcare workers in the control group and 2% in the hydroxychloroquine group, a hazard ratio of 0.2, for a significance level of 5%, statistical power of 90% and assuming a rate to lost-to-follow-up of 10%, a total of 440 subjects is required, 220 per group. (9)

An intention-to-treat analysis will be conducted, with all patients fulfilling inclusion criteria and without exclusion criteria that have had at least one follow-up visit will be included in the analysis. Participants will be censored when they are lost to follow-up (i.e. follow-up visits are not conducted), when they stop the intervention treatment or when they fulfill the primary endpoint.

### 6.2. Data management

All clinical, epidemiological, microbiological and radiological data will be entered in the case report form (CRF) (Appendix 2) and introduced in a centralized database protected with username and password (using OpenClinica online database). For the statistical analysis software Stata 15 or R will be used.

### 6.3. Descriptive analysis

Descriptive analysis will be done for all healthcare workers with at least one follow-up visit. Categorical variables will be expressed as absolute frequency and percentage. Continuous variables will be expressed as mean and standard deviation (SD) or median and interquartile range (IQR). One sample Kolmogorov–Smirnov tests will be performed to assess whether variables were distributed normally.
The following information will be recorded directly on the CRF and will be considered source data: demography, household co-habitants, risk factors and concomitant medication.

### 6.4. Analytical statistics

- Bivariate analysis: Normally distributed numeric parameters will be compared between groups using the t-test or ANOVA. Mann–Whitney U-test or Kruskal–Wallis tests will be used for non-normal variables. Categorical variables will be compared between groups using the Pearson's chi-squared test or Fisher's exact test if the expected frequency is < 5.
- Multivariate analysis:
  - Cox regression models for investigating the association between time to SARS-CoV-2 infection (defined as symptoms compatible with COVID-19 and/or a positive PCR for SARS-CoV-2) ) in the PrEP group compared to placebo group at any time during the 6 months of the follow-up, adjusted for age, sex, concomitant medication, concomitant diseases, laboratory parameters, number of weeks during which the participant was taking care of covid19 patients.

- Cox regression models for investigating the association between time to SARS-CoV-2 seroconversion (defined as IgM or IgG positive) in the PrEP group, compared to placebo in during 6 months of follow-up, adjusted for age, sex, concomitant medication, concomitant diseases, laboratory parameters, number of weeks during which the participant was taking care of covid19 patients.
- Linear regression models for investigating the association between quantitative PCR and treatment group, adjusted for age, sex, concomitant medication, concomitant diseases, laboratory parameters, number of weeks during which the participant was taking care of covid19 patients.

Hierarchical testing will be considered for secondary analysis. Once the primary objective will be established, secondary analyses will be performed.

Interim analyses will be performed monthly and overall type I error will be controlled (see section 8).

## 7- ETHICS AND LEGAL ASPECTS

### 7.1 Regulatory requirements

The study will be performed according to the Declaration of Helsinki (version of Fortaleza, Brazil, October 2013), current ICH-GCP guidelines and all applicable national and local regulatory requirements (Spanish Royal Decree 1090/2015).

### 7.2 Ethical Requirements

This study will be conducted under the auspices of a properly constituted Ethics Committee as defined by ICH-GCP E6 (R2) Guidelines and applicable regulations. This committee will review and approve the final study protocol and subsequent amendments, the final version of the informed consent form and any amendment and any other written information/material to be provided to the participants. Prior to initiation of clinical activity, investigators will provide ISGlobal with a copy of the relevant Ethics Committee approval.

The investigators will follow the indications at the Ethics Committees for Clinical Research at the Adoption of the PRBB Code of GSP and Addendums.

If applicable, the investigators will be responsible for obtaining annual re-approval from the Ethics Committee.

### 7.3 Informed Consent

Informed consent will be signed by all participants before their inclusion in the study. The study consent form will reflect the risks and benefits of participating in the study, and the specific sampling procedures to be done to each participant. Sufficient time will be given to

the participant's guardians to decide whether or not to participate in the study. Candidates will be given the opportunity to enquire about the details of the study and any question regarding the study will be answered.

Participation in this study will be voluntary, and under no circumstances the clinical management of the participants will be affected by the decision to participate or not in the study. The participant will be free to withdraw at any time of the study.

### 7.4 Participant confidentiality

According to the Ley Orgánica 3/2018, del 5 de diciembre de Protección de Datos Personales y Garantía de los derechos digitales; and the Reglamento UE 2016/679 del Parlamento Europeo y del Consejo del 27 de abril de 2016, all information resulting from participating in the study will be confidentially treated. All data and samples collected will be allocated a study code so that they are unidentifiable. No use of any link to the participant's identity will be done. Key identifying information will be kept in locked cabinets away from the coded samples and data and may only be accessed by the study investigators.

Management of patients diagnosed with COVID-19 will be done following current National and Hospital protocols. Declaration of new confirmed COVID-19 cases to Agència de Salut Pública de Barcelona, will be done as per normal practice. Hospital Clinic Director will be also informed of any new case of COVID-19, following current Hospital protocols. Samples obtained under the umbrella of the specific consent form will only be utilized for the purposes of this protocol, and under no circumstances will be used for other purposes as those specified both in the protocol and the consent form. The PIs will ensure that the written information and the consent form are revised when an amendment to the study protocol is made and approved.

A Medical doctor will be available by phone 24 hours during the study period. All participants will be allowed to call this number in case of presenting any adverse event or worsening of symptoms.

Contact telephone numbers:

- From Monday to Friday, 8:30-17:00h: 610584612
- From Monday to Friday (17:00-8:30h) and weekends: 659106155

## 8- SAFETY ASSESSMENT

The principles of ICH GCP require that specific procedures are set to notify and report adverse events in clinical trials. This is a responsibility of the Sponsor and the study physician at each site.

## 8.1. Adverse events and serious adverse events:

### 8.1.1 Definition of Adverse Event (AE)

Adverse event means any untoward medical occurrence associated with the use of an intervention in humans, whether or not considered intervention-related.

Thus, any undesirable medical occurrence in a participant including those events which do not necessarily have a causal relationship to the study drug regimen. Prior to the administration of study drug, only adverse events that meet the definition of serious (see below) and adverse events that the study physician considers to be related to study design and/or procedures should be recorded.

### 8.1.2 Definition of Serious Adverse Event (SAE)

Any untoward medical occurrence that at any dose:

- results in death
- is a life-threatening adverse event
- requires inpatient hospitalization or prolongation of existing hospitalization
- results in a persistent or significant disability/incapacity or substantial disruption of the ability to conduct normal life functions
- or a congenital anomaly/birth defect.

Important medical events that may not result in death, be life-threatening, or require hospitalization may be considered serious when, based upon appropriate medical judgment, they may jeopardize the participant and may require medical or surgical intervention to prevent one of the outcomes listed in this definition.

### 8.1.3 Classification of an Adverse Event

#### 8.1.3.1 Severity of Event

Adverse events will be assessed by direct observations of the study physician or reported by the participant. From the moment a study participant receives the study medication, the study physician will monitor each subject for the development of any clinical evidence of an AE.

The nature of the adverse event, its date and time of onset, duration and severity, therapy employed (if any) and the investigator's opinion of causality to study drug with an alternate aetiology, if appropriate, will be documented. For adverse events to be considered as intermittent or continuous, the events should be of similar nature and severity. The investigator will follow all adverse events to satisfactory clinical resolution or the establishment of a stable chronic stage upon study completion.

The investigator will rate the severity of the adverse event according to the following definition:

| | |
|---|---|
| Mild: | The adverse event is transient and easily tolerated by the subject. |
| Moderate: | The adverse event causes the subject discomfort and interrupts the subject's normal activities. |
| Severe: | The adverse event causes considerable interference with the subject's normal activities, and may be incapacitating or life-threatening. |

The study physician at each site will use the above definitions to assess the relationship of the adverse event to the study drug.

#### 8.1.3.2 Relationship to Study INTERVENTION

The study physician at each site is obligated to assess the relationship between investigational product and the occurrence of each AE/SAE. The study physician will use clinical judgment to determine the relationship. Alternative causes, such as natural history of the underlying diseases, concomitant therapy, other risk factors, and the temporal relationship of the event to the investigational product will be considered.

The degree of certainty about causality will be graded using the categories below.

Probably: An adverse event has a strong temporal relationship to study drug or recurs on re-challenge, and aetiology is unlikely or significantly less likely.

Possibly: An adverse event has a strong temporal relationship to the study drug, and an alternative aetiology is equally or less likely compared to the potential relationship to study drug. The alternate aetiology should be provided by the investigator.

Unlikely: An adverse event has little or no temporal relationship to the study drug and/or a more likely alternative aetiology exists. The alternate aetiology should be provided by the investigator.

Unrelated: An adverse event is due to underlying or concurrent illness or effect of another drug and is not related to the study drug. The alternate aetiology should be provided by the investigator.

#### 8.1.3.3 Expectedness

The study physician will be responsible for determining whether an AE is expected or unexpected. An AE will be considered unexpected if the nature, severity, or frequency of the event is not consistent with the risk information previously described for the study intervention.

### 8.1.4 Time Period and Frequency for Event Assessment and Follow-Up

The occurrence of an AE or SAE may come to the attention of study personnel during study visits and interviews of a study participant presenting for medical care, or upon review by the study monitor.

All AEs including local and systemic reactions not meeting the criteria for SAEs will be captured in the CRF. Information to be collected includes event description, time of onset, the study physician's assessment of severity, relationship to study drug, and time of resolution/stabilization of the event. All AEs that occur during the study duration must be documented appropriately regardless of the relationship. All AEs will be monitored to adequate resolution.

Any medical condition that is present at the time that the participant is screened will be considered as baseline and not reported as an AE. However, if the study participant's condition deteriorates at any time during the study, it will be recorded as an AE.

Changes in the severity of an AE will be documented to allow an assessment of the duration of the event at each level of severity to be performed. AEs characterized as intermittent require documentation of onset and duration of each episode.

### 8.1.5 Adverse Event and serious adverse event Reporting

The study physician at each site will record all events with start dates occurring any time after informed consent is obtained until the last day of study. At each study visit, the study physician will inquire about the occurrence of AE/SAEs since the last visit. Events will be followed for outcome information until resolution or stabilization.

All SAEs will be reported to the Sponsor by the study site physician or an assigned representative within 24 hours of the staff becoming aware of it, using a SAE form, which should be completed, scanned and sent via email to the Sponsor. The SAE form should include nature of event, date of onset, severity, corrective therapies given, outcome and causality (i.e. probably, possibly, unlikely, unrelated). The study physician should assign the causality of the event.

SAE reporting will comply with local regulations for SAE reporting to the sites Research Ethics Committee and/or regulatory authorities. All unexpected SAEs must be reported to the ethics committee and the Spanish regulatory agency (AEMPS) according to Spanish legislation. In addition to the expedite SAE reporting in 24h, monthly aggregate reports will be written by the study physician at each site.

## 9- INTERIM ANALYSIS AND STOPPING RULES

Interim analyses of the efficacy and safety of hydroxychloroquine will be performed monthly and overall type I error will be controlled.

### 9.1. Discontinuation of hydroxychloroquine treatment in participants:

After treatment initiation, hydroxychloroquine will be discontinued in participants presenting severe adverse events related to hydroxychloroquine intake or a confirmed SARS-CoV-2 infection by PCR assay in a nasopharyngeal swab or sputum sample.

All serious adverse events will be notified to the sponsor. The study physician and investigator will evaluate the relationship with the study treatment. Serious and unexpected adverse events that the cause is suspected to be related to hydroxychloroquine will be reported to the sponsor, to the ethical committee, to the Pharmacy Department of the Hospital Clinic of Barcelona and to the Agencia Española del Medicamento y Producto Sanitario (AEMPS).

### 9.2. <u>Stopping rules</u>:

Circumstances that may warrant termination or suspension of the study include:

- Determination of unexpected, significant, or unacceptable risk to participants
- Insufficient compliance to protocol requirements
- Data that are not sufficiently complete and/or evaluable
- Determination that the primary endpoint has been met
- Determination of futility

## 10- STUDY MONITORING

During the study, regular monitoring will be conducted to:
-Confirm adherence to the protocol and applicable guidelines and regulations
-Ensure adequate recording of data
-Ensure adequate handling of biological samples and investigational product
-Verification of informed consent process and withdrawals

## 11- FUNDING

The study will be supported by funds from each clinical trial site and may receive further support from the public or non-for-profit-private sector. Laboratorios Rubió has partially contributed to the funding of this project and with the required doses of hydroxychloroquine (Dolquine ®).

## 12- PUBLISHING POLICY

The results of the study will be published as an original article in an international journal. All co-I will be included as co-authors or in the Acknowledgements of the article.

## 13- REFERENCES:

[1] Chan JF, et al. A familial cluster of pneumonia associated with the 2019 novel coronavirus indicating person-to-person transmission: a study of a family cluster. The Lancet. 2020; 395: 514-523.

[2] Zhu, N. A Novel Coronavirus from Patients with Pneumonia in China, 2019. N Engl J Med. 2020; 382: 727-733.

[3] World Health Organization. Coronavirus disease 2019 (COVID-19). Situation report. https://www.who.int/docs/default-source/coronaviruse/situation-reports/20200313-sitrep-53-covid-19.pdf?sfvrsn=adb3f72_2

[4] Report of the WHO-China Joint Mission on Coronavirus Disease 2019 (COVID-19). 16-24 February 2020.

[5] Chou R, Evans C, Hoverman A, et al. Preexposure Prophylaxis for the Prevention of HIV Infection: Evidence Report and Systematic Review for the US Preventive Services Task Force. *JAMA.* 2019;321(22):2214–2230. doi:10.1001/jama.2019.2591

[6]Wang M, et al. Remdesivir and chloroquine effectively inhibit the recently emerged novel coronavirus (2019-nCoV) in vitro. Cell Research. 2020. doi:10.1038/s41422-020-0282-0.

[7] Yao X, et al. In Vitro Antiviral Activity and Projection of Optimized Dosing Design of Hydroxychloroquine for the Treatment of Severe Acute Respiratory Syndrome Coronavirus 2 (SARS-CoV-2). Clin Infect Dis. 2020 Mar 9. pii: ciaa237. doi: 10.1093/cid/ciaa237

[8] Zhou D, Dai SM, Tong Q. COVID-19: a recommendation to examine the effect of hydroxychloroquine in preventing infection and progression. The Journal of antimicrobial chemotherapy. 2020

[9] Lakatos E, et al. A comparison of sample size methods for the logrank statistic. Statistics in Medicine. 1992;11:179–191.
